# Effect of a 24‐week resistance exercise intervention on cognitive function in cognitively normal older adults: The AGUEDA randomized controlled trial

**DOI:** 10.1002/alz.71019

**Published:** 2026-01-21

**Authors:** Beatriz Fernandez‐Gamez, Patricio Solis‐Urra, Andrea Coca‐Pulido, Cristina Molina‐Hidalgo, Marcos Olvera‐Rojas, Esmée A. Bakker, Darío Bellón, Alessandro Sclafani, Jose Mora‐Gonzalez, Javier Fernández‐Ortega, Lucía Sánchez‐Aranda, Isabel Martín‐Fuentes, Angel Toval, Javier Sanchez‐Martinez, Lu Wan, Manuel Gomez‐Rio, Teresa Liu‐Ambrose, Kirk I. Erickson, Francisco B. Ortega, Irene Esteban‐Cornejo

**Affiliations:** ^1^ Department of Physical Education and Sports Faculty of Sport Sciences Sport and Health University Research Institute (iMUDS) University of Granada Granada Spain; ^2^ AdventHealth Research Institute Neuroscience Institute Orlando Florida USA; ^3^ Faculty of Education and Social Sciences Universidad Andrés Bello Viña del Mar Chile; ^4^ Servicio de Medicina Nuclear Hospital Universitario Virgen de las Nieves Granada Spain; ^5^ Instituto de Investigación Biosanitaria ibs.GRANADA Granada Spain; ^6^ Department of Physical Therapy Faculty of Medicine University of British Columbia Vancouver British Columbia Canada; ^7^ Centre for Aging SMART at Vancouver Coastal Health Vancouver Coastal Health Research Institute Vancouver British Columbia Canada; ^8^ Djavad Mowafaghian Centre for Brain Health Vancouver Coastal Health Research Institute Vancouver British Columbia Canada; ^9^ Centro de Investigación Biomédica en Red Fisiopatología de la Obesidad y Nutrición (CIBERobn) Instituto de Salud Carlos III Madrid Spain; ^10^ Faculty of Sport and Health Sciences University of Jyväskylä Jyväskylä Finland

**Keywords:** aging, brain health, cognition, physical activity, strength training

## Abstract

**INTRODUCTION:**

The Active Gains in Brain Using Exercise During Aging (AGUEDA) trial examined the effects of a 24 week resistance exercise (RE) intervention on executive function (EF) and other cognitive domains in cognitively normal older adults.

**METHODS:**

Ninety participants (mean age, 71.8 years; 57.8% female) were randomized to an RE or control group. At baseline and 24 weeks, EF and other cognitive domains were assessed.

**RESULTS:**

The RE group showed significant improvements in overall EF (standardized mean difference [SMD] = 0.39, 95% confidence interval = 0.14, 0.65), with no significant between‐group difference (SMD = 0.13, *p* = 0.37). The RE group showed a significant improvement in attentional/inhibitory control (SMD = 0.43, *p* < 0.001) compared to the control group, while no effects were observed in other domains (all *p* > 0.12). Moderation by age, education, and subjective cognitive decline was observed.

**DISCUSSION:**

Although no overall EF benefit was observed, RE improved attentional/inhibitory control in cognitively normal older adults. RE may yield greater benefits in vulnerable subgroups.

**CLINICAL TRIAL REGISTRATION:**

The trial was registered on ClinicalTrials.gov (ClinicalTrials.gov Identifier: NCT05186090).

**Highlights:**

- Cognitive effects of resistance exercise (RE) may vary across different cognitive domains in cognitively healthy older adults.
- Twenty‐four week RE produced selective improvements in attention/inhibitory control.
- RE did not improve executive function (EF), or other cognitive domains (episodic memory, processing speed, visuospatial processing, and working memory).
- RE improved muscular strength, which were associated with gains in EF, episodic memory and working memory.
- There is value in personalized exercise interventions tailored to individual risk populations, such as those with higher subjective cognitive decline.

## 1 BACKGROUND

 Dementia is a major cause of disability worldwide,^1^, ^2^, ^3^ representing one of today's most pressing public health challenges. The current lack of disease‐modifying treatments highlights the critical need for preventive strategies aimed at preserving cognitive function.^3^, ^4^, ^5^, ^6^ Exercise is one of the most promising non‐pharmaceutical interventions for the maintenance and/or enhancement of cognitive function in late life.^4^, ^5^, ^7^ Interest in resistance exercise (RE) is growing rapidly, as RE may have a positive effect on cognitive function,^8^ and can serve as a valuable strategy for preventing dementia,^9^ much like other types of exercise.^4^, ^10^, ^11^ Recent evidence suggested that RE may provide specific cognitive benefits, particularly in domains such as executive function (EF) and memory,^12^ possibly through enhanced activation of brain regions involved in these processes.^13^ Additionally, RE using elastic bands or body weight provide a practical, affordable, and accessible alternative to conventional machine‐based or free‐weight resistance training, which is supported by a growing body of evidence showing its positive effects on cognitive health.^14^, ^15^, ^16^

There is considerable variability in how cognitive performance is measured across studies, making comparisons difficult. Prior exercise interventions on the cognitive effects of elastic‐band and body‐weight RE have mainly relied on global cognitive screening measures (e.g., Montreal Cognitive Assessment [MoCA] and Mini‐Mental State Examination [MMSE]),^17^, ^18^ or single cognitive tests to measure a domain (e.g., Rey Auditory Verbal Learning Test for episodic memory or the Trail Making Test [TMT] for attention^19^). These approaches may not fully capture the complexity of cognitive function, and only a limited number of studies have used comprehensive batteries designed to assess distinct cognitive domains, such as EF,^19^ attention,^20^ processing speed,^21^ or memory.^22^

**RESEARCH IN CONTEXT**

1. **Systematic review**: We conducted a systematic search in PubMed, Web of Science, and Scopus to identify randomized controlled trials (RCT) investigating the effects of resistance exercise (RE) on cognitive function in older adults. Search terms combined expressions like “resistance training,” “strength training,” “cognitive function,” and “aging.”
2. **Interpretation**: RE did not lead to improvements in executive function overall or in other cognitive domains, including episodic memory, processing speed, visuospatial processing, and working memory; however, it produced selective improvements in attentional/inhibitory control. Cognitive effects appeared to be particularly pronounced in vulnerable subgroups, such as individuals reporting memory complaints.
3. **Future directions**: These findings align with existing evidence suggesting that the cognitive effects of RE may vary across different cognitive domains and be influenced by individual participant's characteristics. This supports the value of personalized exercise interventions tailored to individual risk populations.

In this context, while EF has been a key target in exercise‐cognition research, recent reviews suggest that RE may not affect all cognitive domains equally.^23^ This underscores the need to identify the specific cognitive domains that are most responsive to exercise‐related improvements, as well as to determine which individuals are most likely to benefit from these interventions.^24^ Hence, it remains unclear whether RE, particularly when performed with elastic bands, exerts broad effects across global cognitive function or whether the benefits are domain specific.^25^

Individual demographic or genetic characteristics, such as apolipoprotein E (*APOE*) ε4,^26^ age,^27^ or subjective cognitive decline (SCD)^28^ may also moderate the effects of RE on cognition in older adults.^29^ Previous studies with aerobic exercise have reported greater cognitive effect in males^30^ and *APOE* ε4 carriers.^31^ Interestingly, studies with RE interventions found greater cognitive benefits in females,^32^ *APOE* ε4 non‐carriers,^33^ in oldest participants,^34^ higher SCD,^35^ and older adults with lower education level.^36^ Thus, these findings underscore the complexity of understanding the role of individuals’ characteristics in moderating the cognitive effects of exercise, and specifically of RE.

Well‐designed randomized controlled trials (RCTs) examining the effects of easily prescribed RE interventions, along with the role of potential moderators, are crucial for advancing our understanding of how to optimize cognitive function in older adults. These findings could contribute to public health guidelines by providing tailored exercise recommendations for preserving cognitive health in older adults. The Active Gains in Brain Using Exercise During Aging (AGUEDA) trial was designed to investigate the effects of a 24‐week RE intervention using elastic bands and body weight on cognitive function in cognitively normal older adults. The primary outcome was an EF composite score, whereas secondary outcomes included other cognitive domains (i.e., attentional/inhibitory control, episodic memory, processing speed, visuospatial processing, and working memory). Second, we examined the effects of several individual‐level moderators (i.e., sex, age, education level, comorbidities, *APOE* ε4 carriership, amyloid burden, baseline cognitive performance, and baseline SCD). Third, we explored the effects of RE on physical condition parameters (i.e., muscular strength, physical function, and cardiorespiratory fitness). Finally, we explored whether any of these exercise‐related outcomes statistically mediated the exercise‐derived cognitive improvements. The overall hypothesis was that a 24 week RE intervention would improve selective cognitive domains in cognitively normal older adults, particularly in the more vulnerable subgroups (e.g., the oldest participants, those with SCD or with fewer years of education), and these cognitive benefits would be mediated by improvements in muscular strength.

## 2 MATERIALS AND METHODS

### 2.1 Design and participants

The AGUEDA trial was a single‐center, two‐arm, single‐blind RCT that randomized 90 cognitively normal older adults (65–80 years old) from Granada, Spain. All data were collected between March 2021 and May 2022. The trial protocol followed the principles of the Declaration of Helsinki and was approved by the research ethics board of the Andalusian Health Service (CEIM/CEI Provincial de Granada; #2317‐N‐19) on May 25, 2020. The trial was registered on ClinicalTrials.gov (ClinicalTrials.gov Identifier: NCT05186090). All participants provided written informed consent. The reporting of the results adheres to the Consolidated Standards of Reporting Trials Extension (CONSORT extension) guidelines (see Supplementary Material 1, Table S1 in supporting information). All outcome‐related measures and analyses were performed by staff who were blinded to the intervention assignment. Details of the AGUEDA project's methodology and protocols have been described elsewhere^37^ and deposited at GitHub.^38^

### 2.2 Randomization, masking, and eligibility criteria

Randomization occurred on a rolling basis and only after the completion of all baseline assessment sessions by each participant, to reduce the risk of bias during the assessment. Stratification by two factors was considered: age at study entry (≤ 72, > 72 years) and sex (male, female). Eligibility criteria were defined as: (1) older adults between 65 and 80 years old; (2) physically inactive (i.e., defined as not participating in any RE program in the last 6 months, and accumulating < 600 metabolic equivalents of task minutes/week by the International Physical Activity Questionnaire [IPAQ]);^39^ (3) cognitively normal according to the Spanish version of the modified Telephone Interview of Cognitive Status (STICS‐m; ≥ 26/41 points), MMSE (≥ 25/30), and MoCA (< 71 years: ≥ 24/30; 71–75 years: 22/30; > 75 years: 21/30); and (4) not to present significant depressive symptoms at baseline according to the Geriatric Depression Scale (GDS; ≥ 15). Detailed information about eligibility criteria is detailed in Supplementary Material 2, Table S2 in supporting information, and available elsewhere.^37^

### 2.3 Intervention and control

Details of the methodology of the AGUEDA exercise intervention have been fully described elsewhere following the Consensus on Exercise Reporting Template (CERT).^40^ Briefly, the participants randomly assigned to the 24 week RE intervention group were asked to attend three sessions per week, for 60 minutes each day (10 minutes for the warm‐up phase, 45 minutes for the main phase, and 5 minutes for the cool‐down phase) at the Sport and Health University Research Institute research center (iMUDS), in Granada, Spain. The RE intervention consisted of a combination of upper and lower limb exercises using elastic bands and body weight. The volume and intensity were based on the resistance of the elastic bands (TheraBand; seven resistances divided by colors),^41^ number of repetitions (individualized), motor complexity of exercises (three levels), sets and rest (three sets/60 seconds rest), execution time (40–60 seconds), and velocity (as fast as possible). The groups were asked to maintain their usual lifestyle, and no additional activities were encouraged. After the 24 week period, the control group was offered the RE intervention for ethical reasons. The intervention description follows the Template for Intervention Description and Replication (TIDieR) guidelines (Supplementary Material 3, Table S3 in supporting information).

### 2.4 Power calculation and sample size

The power calculation was based on a meta‐analysis which found the effect size of exercise interventions on the EF composite score in older adults to be 0.34, with a 95% confidence interval of 0.22 to 0.47,^11^ with a two‐tailed alpha of 0.05, and a power of 80%. After adjusting for a 20% estimated dropout rate, 45 participants for each group were needed for sufficient power.

### 2.5 Outcomes measurements

The primary outcome (EF composite score) was assessed at baseline, midpoint (12 weeks), and post‐intervention (24 weeks), whereas secondary and other outcomes were assessed only at baseline and post‐intervention. A summary of the cognitive tests used is presented in Table 1, while detailed descriptions of all outcome measures can be found in Supplementary Material 4, 5, and 6.

**TABLE 1:** Summary of cognitive measures and outcomes included in each cognitive domain.

Domain	Cognitive test	Outcome (unit)
Composite executive function score	Trail Making Test (TMT)	Interference score (Time part B – Time part A) (sec)
	Digit Symbol Substitution Test (DSST)	Total correct score (n)
	Dimensional Change Card Sort Test (DCCST)	Inverse efficiency score (switch trials ‐ high load) (rt/acc)
	Spatial Working Memory Test (SWMT)	Inverse efficiency score of all trials (rt/acc)
Attentional/inhibitory control	Trail Making Test (TMT)	Time part B (sec)
	Dimensional Change Card Sort Test (DCCST)	Computed score* (n)
	Stroop Test	Incongruent raw score (rt)
	Flanker Test	Computed score* (n)
Episodic memory	MoCA delayed recall	Multiple choice cue (n)
	Picture Sequence Memory Test (PSMT)	Raw score (n)
	Rey Auditory Verbal Learning Test (RAVLT)	Total recall raw score – sum of the 5 consecutive learning trials from List A (n)
		Total delayed recall correct raw score – sum of words of List A after 20 minutes (n)
		Recognition – number of correct responses of the recognition trial – number of intrusions false correct responses of the recognition trial) (n)
	Rey ‐ Osterrieth Complex Figure Test (ROFT)	Raw score copy (n)
		Delayed raw score after 3 minute period – total of points (n)
Processing speed	Digit Symbol Substitution Test (DSST)	Total correct raw score (n)
	Trail Making Test (TMT)	Time part A (sec)
Visuospatial processing	Wechsler Adult Intelligence Scale	Block design (acc)
		Matrix reasoning (acc)
	MoCA clock draw	Total, score (n)
Working memory	Spatial Working Memory Test (SWMT)	3‐item (acc)
		4‐item (acc)
	List Sorting Working Memory (LSWMT)	Total correct raw score (n)
	N‐back	2‐back (acc)

The Trail Making Test, Dimensional Change Card Sort Test, Spatial Working Memory Test, and Stroop Test variables were reverted in the analyses so that higher values indicate better performance. Pl Computed score: combines accuracy and reaction time (range: 0–10). If accuracy ≤ 80%, the score equals accuracy; if accuracy > 80%, the score is a combination of both metrics.

Abbreviations: acc, accuracy; n, number; rt, reaction time; sec, seconds; SD: standard deviation.

#### 2.5.1 Primary outcome: EF

An EF composite score was created by performing confirmatory factor analyses (CFA) (Supplementary Material 4, 2. EF score). The cognitive tests included in the CFA were prespecified in the methodology of the AGUEDA trial^37^ based on available evidence and theoretical positions of EF.^42^ A first‐order factor CFA was performed for the final EF composite score with the following cognitive indicators: TMT (Interference Score, Time part B – Time part A), Digit Symbol Substitution test (DSST; total correct score), Dimensional Change Card Sort Test (DCCST; inverse efficiency score, reaction time/accuracy), and Spatial Working Memory Test (SWMT; inverse efficiency score of switch trials – high load, reaction time/accuracy).

Other tests were included in the first‐order factor CFA but were not retained in the final EF composite score (i.e., Picture Sequence Memory Test [PSMT], List Sorting Working Memory Test [LSWMT], Flanker test, Stroop test, and task switching test). The methodological details and results of the CFA are provided in Supplementary Material 4 (Tables S4–S7 and Figures S1–S2 in supporting information).

#### 2.5.2 Secondary outcomes: other cognitive domains—attentional/inhibitory control, episodic memory, processing speed, visuospatial processing, and working memory

Participants completed a comprehensive neuropsychological evaluation that measured different cognitive domains detailed in Table 1 and Supplementary Material 4; Table S8 in supporting information):^43^

1. Attentional/Inhibitory control: TMT (Time part B), DCCST (computed score), Stroop test (incongruent raw score), and Flanker test (computed score).
2. Episodic memory: MoCA delayed recall (multiple choice cue), PSMT (raw score), Rey Auditory Verbal Learning Test (total recall, delayed, and recognition raw score), Rey–Osterrieth Complex Figure Test (ROFT; copy and delayed raw score).
3. Processing speed: DSST (total correct raw score) and TMT (Time part A).
4. Visuospatial processing: Wechsler Adult Intelligence Scale with Matrix Reasoning and Block Design (accuracy) and MoCA Clock Draw (total score).
5. Working memory: SWMT (3 & 4 items), LSWMT (total correct), N‐Back Test (2‐back).

#### 2.5.3 Moderation outcomes

Sociodemographic and AD‐related related variables were examined as potential moderators, as detailed in Supplementary Material 6. These included: (1) sex assigned at birth (male vs. female); (2) age (≤ 72 years old vs. > 72 years); (3) education (< 12 years vs. ≥ 12 years of education); (4) number of comorbidities (< 3 vs. ≥ 3), including conditions related to hypertension, diabetes, heart disease, obesity, and cholesterol; (5) *APOE* ε4 carrier (carrier vs. non‐carrier); (6) amyloid burden (positive vs. negative, 12 Centiloid [CL]); (6) baseline cognitive performance of each domain (< median vs. ≥ median); and (7) median baseline SCD (< 3 points vs. ≥ 3 points).

#### 2.5.4 Physical condition parameters: muscular strength, physical function, and cardiorespiratory fitness

Participants completed a battery of physical tests to evaluate muscular strength, physical function, and cardiorespiratory fitness (See Supplementary Material 5). (1) Muscular strength included both upper and lower body muscular strength. Upper body muscular strength was assessed using the arm curl test from the Senior Fitness Test (SFT), handgrip strength with a hand dynamometer (TKK5101 Grip D, Takey), and elbow extension strength with the Gymmex Iso‐2 dynamometer (EASYTRCH s.r.l.). Lower body strength was evaluated using the 30 second sit‐to‐stand test from SFT, the five times sit‐to‐stand test from Short Physical Performance Battery (SPPB), and knee extension strength measured with the Gymmex Iso‐2 dynamometer (EASYTRCH s.r.l.). (2) Physical function was assessed by the 2 minute step test and the up and go test, from the SFT. (3) Cardiorespiratory fitness was assessed by the 6 minute walk test and the 2 km walking test. All individual test results were standardized into *z* scores for each variable using the baseline mean and standard deviation (SD). A composite score was then calculated as the mean of the corresponding *z* scores for each domain (muscular strength, physical function, and cardiorespiratory fitness). These averages were further standardized into a *z* score, yielding a final composite score with a mean of 0 and a SD of 1, which facilitates interpretation and comparison across domains. The raw values are provided in Table S9.

### 2.6 Intervention attendance and intensity

Session attendance was calculated as the proportion of sessions completed out of the total prescribed sessions (i.e., 72 sessions scheduled over 24 weeks at a frequency of three sessions per week).^44^ For the per‐protocol (PP) analysis, 80% attendance (i.e., > 57 exercise sessions) was required. Any missed session was registered and rescheduled to an alternative date to maximize intervention attendance. Intensity was measured using the 10‐point Borg Rating of Perceived Exertion scale (RPE) ^45^ with a target rating from 4 to 8 depending on the prescribed intensity level for each week.

### 2.7 Adverse events

All adverse events (AEs) were reviewed and recorded in REDCap,^46^ preferably at the time of the event; however, most of them were collected retrospectively at the end of the intervention period, based on participant self‐report and review of clinical records when necessary. AEs were defined and categorized according to the Common Terminology Criteria for Adverse Events (CTCAE v4.0),^47^ using standardized definitions and severity grading (grade 1 = mild, grade 2 = moderate, grade 3 = severe, grade 4 = life threatening, and grade 5 = death;^48^ see Supplementary Material 7, Table S10 in supporting information for categorization). AEs were initially reviewed by the research staff, and a medical professional adjudicated the classification and relatedness to the intervention in cases of uncertainty.

### 2.8 Statistical analysis

#### 2.8.1 Main analysis

The main analyses were based on both available‐case intention‐to‐treat (ITT), which included all randomized participants, and PP, which included participants attending at least 80% of sessions (> 57 sessions).

Data preprocessing involved reviewing the data's biological plausibility to identify implausible values resulting from suboptimal testing conditions. The 12 week and 24 week outcomes *z* scores were calculated using the baseline mean and SD for each variable. The use of these *z* score values in whole‐sample analyses allows comparisons across outcomes of different natures, and the *z* score of change (i.e., post‐intervention *z* score) can be interpreted as a standardized effect size because it indicates how many SDs the outcomes at post‐intervention have changed with respect to the baseline mean and SD.^49^ For sensitivity analyses, winsorization was applied to the raw scores of each outcome to reduce the influence of extreme values (i.e., scores ± 4 SD from the mean). Extreme values were replaced with the nearest value within the acceptable range.^34^

Intervention effects were evaluated by examining between‐group differences (exercise vs. control) from baseline to 24 weeks in EF, other cognitive domains (i.e., attentional/inhibitory control, episodic memory, processing speed, visuospatial processing, and working memory), and physical condition parameters (i.e., muscular strength, physical function, and cardiorespiratory fitness) following constrained linear mixed models (CLMM; i.e., baseline adjusted), specifically with LMMstar and lmerTest packages in R Studio,^49^ with restricted maximum likelihood estimation. The models included time and group as a categorical fixed effect, and group‐by‐time interactions, with the intercept specified as a random effect. Unequal variance was allowed across time and group. Statistical significance was set at *p* < 0.05 without adjustments for multiple comparisons because the primary analysis was based on baseline and 24‐week comparisons. Estimated marginal means, within‐group differences, and between‐group differences were calculated by emmeans package. The intervention effect was represented by the coefficient for the interaction term in the model, along with its (95% confidence interval [CI]). Analyses were carried out using R, version 4.5.0.

#### 2.8.2 Subgroup analysis

Exploratory subgroup analyses were conducted to examine interindividual variability in cognitive responses and to identify specific subgroups that may respond differently to the intervention, potentially offering insights that could inform future research and the development of more individualized intervention strategies. Interaction terms were included, and stratified analyses were performed to examine whether key variables (i.e., sex, age, education level, comorbidities, *APOE* ε4 carriership, amyloid burden, baseline cognitive performance, and SCD) modified the intervention's effect on EF and other cognitive domains. The models followed the same statistical approach as in the primary analysis, using CLMM, specifically using the LMMstar and lme4 packages^49^ with restricted maximum likelihood estimation. All interactions were interpreted as *p* < 0.1. A false discovery rate (FDR) correction was applied (16 comparations per outcome) as a sensitivity analysis using the Benjamini–Hochberg approach, via the p.adjust function in R.

#### 2.8.3 Post hoc mediation analysis

Mediation analyses were conducted according to the Guideline for Reporting Mediation Analyses (AGReMA) using the lme4 and mediation packages in R.^50^ Muscular strength, physical function, and cardiorespiratory fitness indicators were proposed as potential mediators of the effects of exercise on EF and other cognitive domains. Only the indicators showing significant effects were assessed to understand the mechanisms through which the intervention could exert its effect. Standardized (β) regression coefficients are presented for four equations. Equation 1 regresses the mediator (e.g., muscular strength) on the independent variable (exercise vs. control). Equation 2 regresses the dependent variables (i.e., cognitive domain) on the independent variable. Equation 3 regresses the dependent variables on the mediator (equation 3) and the independent variable (equation 3'). The outcome of interest at baseline was included as a covariate. The model's statistical significance was assessed with percentile bootstrapping (*n* = 10.000) and 95% CIs. Outcomes are considered statistically significant when CIs do not include zero. To determine how much of the total effect was explained by the mediation, the percentage of the total effect was computed as follows: (indirect effect / total effect) × 100.

#### 2.8.4 Adverse event

A sensitivity analysis was performed using an ITT principle, including all randomized participants, except for those who experienced AEs demonstrated to influence the primary outcomes (e.g., stroke^50^ and cancer^51^).

## 3 RESULTS

### 3.1 Sample characteristics

**FIGURE 1:**
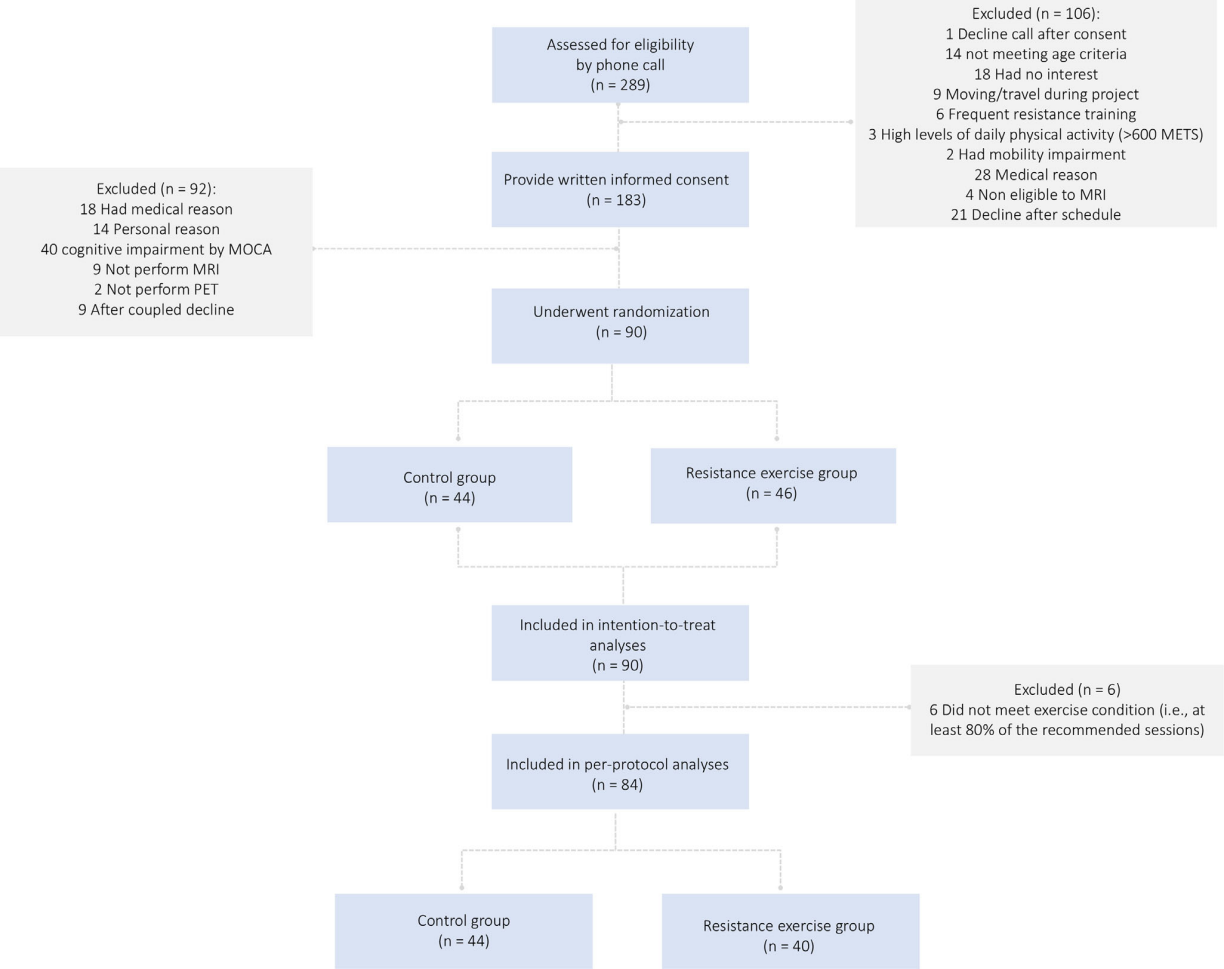
Consolidated Standards of Reporting Trials (CONSORT) flow diagram of the Active Gains in Brain Using Exercise During Aging (AGUEDA) trial. METS, metabolic equivalents of task; MoCA, Montreal Cognitive Assessment; MRI, magnetic resonance imaging; PET, positron emission tomography.

A total of 289 participants were assessed for eligibility by phone call. Of these, 183 provided consent to participate and 90 participants were randomized (recruitment rate = 31.1%; Figure 1 shows the flowchart with specific numbers and reason of exclusion). A total of 10 out of 90 participants dropped out of the study, resulting in an overall attrition rate of 11.1% (6.5% [3/46] in the RE group and 15.9% [7/44] in the control group). Table 2 shows the participants’ demographic characteristics. The mean age at baseline was 71.75 ± 3.96 years, and 52 (57.8%) females were included in the study. The mean education level was 11.54 ± 4.90 years. Among the participants, 13 (14.8%) were classified as *APOE* ε4 carriers and 19 (21.1%) as having positive amyloid burden. In addition, 37 participants (41.1%) had 3 or more comorbidities, and the mean score for SCD was 2.92 ± 1.97.

**TABLE 2:** Baseline descriptive characteristics of the Active Gains in Brain Using Exercise During Aging (AGUEDA) sample.

	All	Exercise group	Control group
	Mean ± SD	Mean ± SD	Mean ± SD
Overall characteristics			
	90	46	44
Female, no. (%)	52 (57.8)	27 (58.7)	25 (56.8)
Age at baseline, yr	71.75 (3.96)	71.90 (4.21)	71.58 (3.73)
Education length, yr	11.54 (4.90)	11.17 (5.26)	11.93 (4.53)
12 years of education or less, *n* (%)	57 (63.3)	32 (69.6)	25 (56.8)
Height, cm	160.44 (8.98)	160.17 (8.87)	160.72 (9.18)
Weight, kg	73.50 (12.86)	72.89 (13.41)	74.14 (12.39)
Body mass index, kg/m^2^	28.50 (4.23)	28.35 (4.43)	28.67 (4.05)
3 or more comorbidities, n (%)	37 (41.1)	15 (32.6)	22 (50.0)
*APOE* ε4 carrier, n (%)	13 (14.8)	7 (15.6)	6 (14.0)
Amyloid positive, n (%)	19 (21.1)	8 (17.4)	11 (25.0)
Subjective cognitive decline, score	2.92 (1.97)	2.74 (1.99)	3.11 (1.94)
Cognitive status			
Telephone Interview of Cognitive Status, score	33.91 (2.72)	33.78 (2.65)	34.05 (2.81)
Mini‑Mental State Examination, score	28.92 (1.06)	29.00 (0.97)	28.84 (1.16)
Montreal Cognitive Assessment, score	25.58 (2.14)	25.28 (2.21)	25.89 (2.05)
Cognitive function			
Executive function, *z* score	−0.01 (1.00)	−0.03 (1.07)	0.01 (0.94)
Episodic memory, *z* score	0.00 (1.01)	−0.01 (0.85)	0.01 (1.15)
Processing speed, *z* score	−0.01 (1.00)	0.04 (0.85)	−0.06 (1.15)
Working memory, *z* score	−0.02 (0.99)	0.01 (1.05)	−0.04 (0.94)
Attentional/inhibitory control, *z* score	−0.01 (1.00)	0.04 (0.98)	−0.06 (1.03)
Visuospatial processing, *z* score	−0.01 (1.00)	−0.09 (0.94)	0.08 (1.06)
Physical condition parameters			
Muscular strength, *z* score	0.00 (1.03)	0.08 (1.03)	−0.07 (0.98)
Physical function, *z* score	0.00 (1.00)	−0.01 (0.96)	0.01 (1.04)
Cardiorespiratory fitness, *z* score	0.00 (1.00)	0.02 (0.95)	0.01 (1.06)

*Reverted variable (*‐1) for easy interpretation, the higher score, the better performance.

Abbreviations: *APOE*, apolipoprotein E; m, meters; n, number; SD, standard deviation; yr, years.

### 3.2 Primary outcome

**FIGURE 2:**
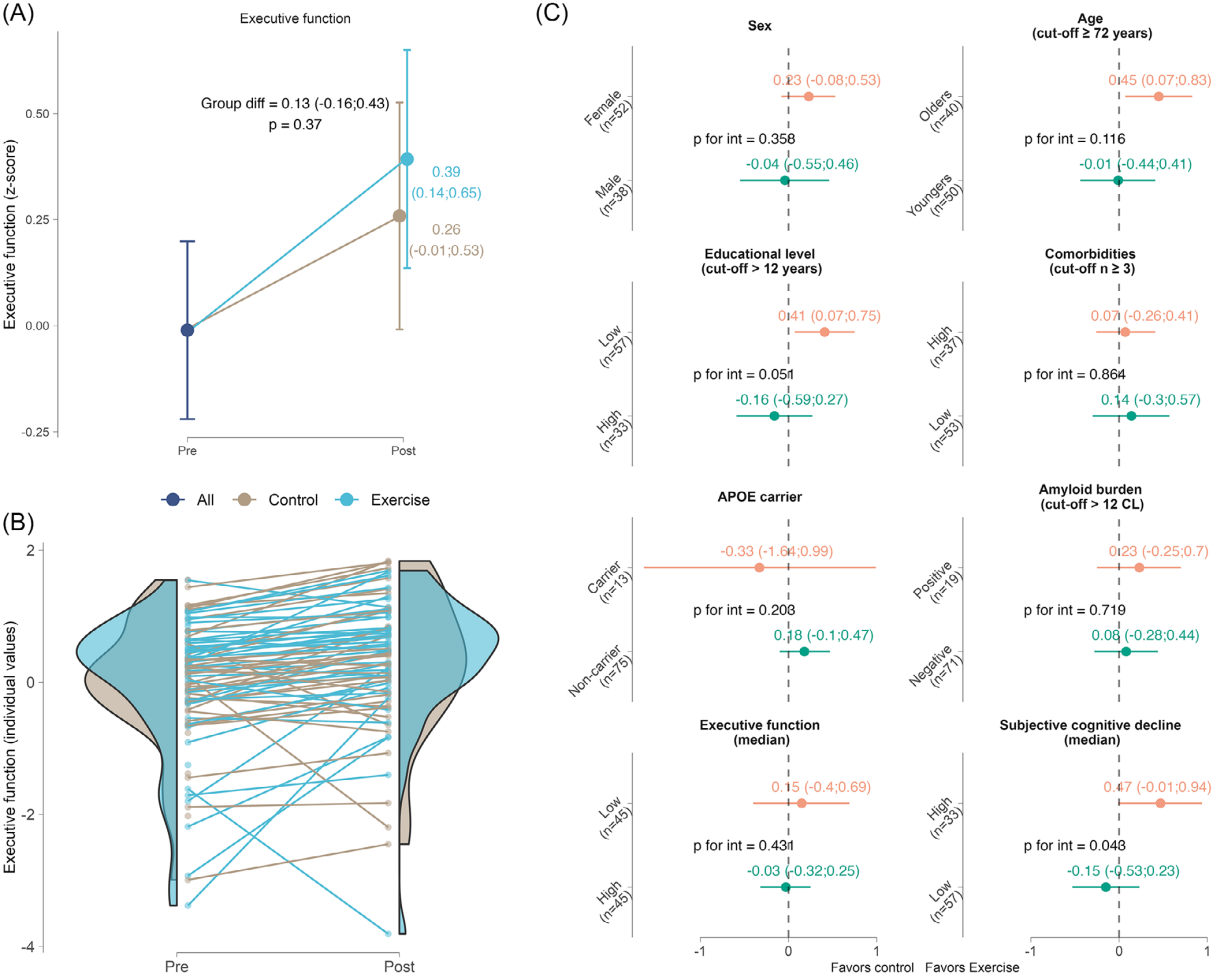
Intention‐to‐treat effects of the 24 ‐week resistance exercise intervention on executive function for the whole sample (A–mean values; B–individual values) and for subgroups (C). A, Dots indicate estimated marginal means at each time point (and 95% confidence intervals). B, Dots indicate individual raw values. C, Dots indicate the mean difference of marginal means and 95% confidence intervals between the exercise and control groups in each subgroup

The RE group showed significant improvements on the EF composite score at 24 weeks (standardized mean difference [SMD] = 0.39, 95% CI: 0.14, 0.65), whereas the control group did not (SMD = 0.26, 95% CI: −0.01, 0.53). However, no significant between‐group differences were detected (SMD = 0.13, P(*p* = 0.37); Figure 2A, 2B).

### 3.3 Secondary outcomes

**FIGURE 3:**
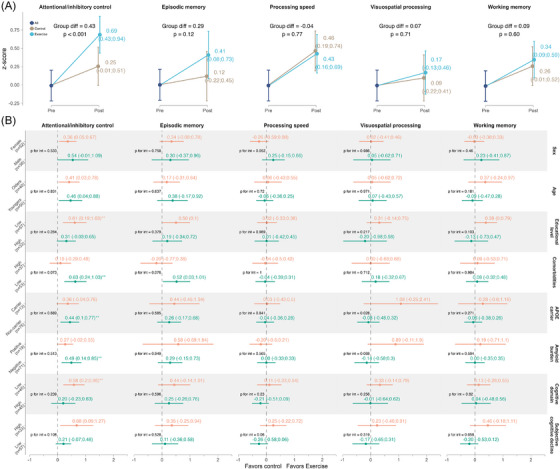
Intention‐to‐treat effects of the 24 week resistance exercise intervention on cognitive domains for the whole sample (A) and subgroups (B). A, Dots indicate the estimated marginal means at each time point (and 95% confidence intervals). B, Dots indicate the mean difference of the marginal means and 95% confidence intervals between exercise and control in each subgroup.**Indicated that the *p* value survived the false discovery rate correction for multiple testing. *APOE*, apolipoprotein E

The RE group demonstrated significantly greater improvements in attentional/inhibitory control than the control group at 24 weeks (SMD = 0.43, *p* < 0.001). No significant between‐group differences were detected in episodic memory (SMD = 0.29, *p* = 0.12), processing speed (SMD = −0.04, *p* = 0.77), visuospatial processing (SMD = 0.07, *p* = 0.71), or working memory (SMD = 0.09, *p* = 0.60) at 24 weeks (Figure 3A).

### 3.4 Moderation analysis

Differences in RE effects on EF (Figure 2C and Supplementary Material 7, Table S11 in supporting information) and other cognitive domains (Figure 3B and Supplementary Material 7, Tables S12 and S13 in supporting information) were examined with moderators at baseline (sex, age, education level, comorbidities, *APOE* ε4 carriership, amyloid burden, baseline cognitive performance, and SCD). Greater effects on EF were found for participants reporting higher baseline levels of SCD (SMD = 0.47, 95% CI: −0.01, 0.94), compared to those reporting lower levels (SMD = −0.15, 95% CI: −0.53, 0.23; *p* for interaction = 0.043), for those with lower education level (SMD = 0.41, 95% CI: 0.07, 0.75), compared to those reporting higher education level (SMD = −0.16, 95% CI, −0.59, 0.27; *p* for interaction = 0.05), and for the older participants (SMD = 0.45, 95% CI: 0.07, 0.83), compared to younger ones (SMD = −0.01, 95% CI: −0.44, 0.41; *p* for interaction = 0.11). Regarding the other cognitive domains, trends toward moderation were observed. RE consistently showed a trending moderation by baseline SCD in three out of the five cognitive domains: attentional/inhibitory control (*p* = 0.10), processing speed (*p* = 0.06), and working memory (*p* = 0.05).

### 3.5 Physical condition parameters

**FIGURE 4:**
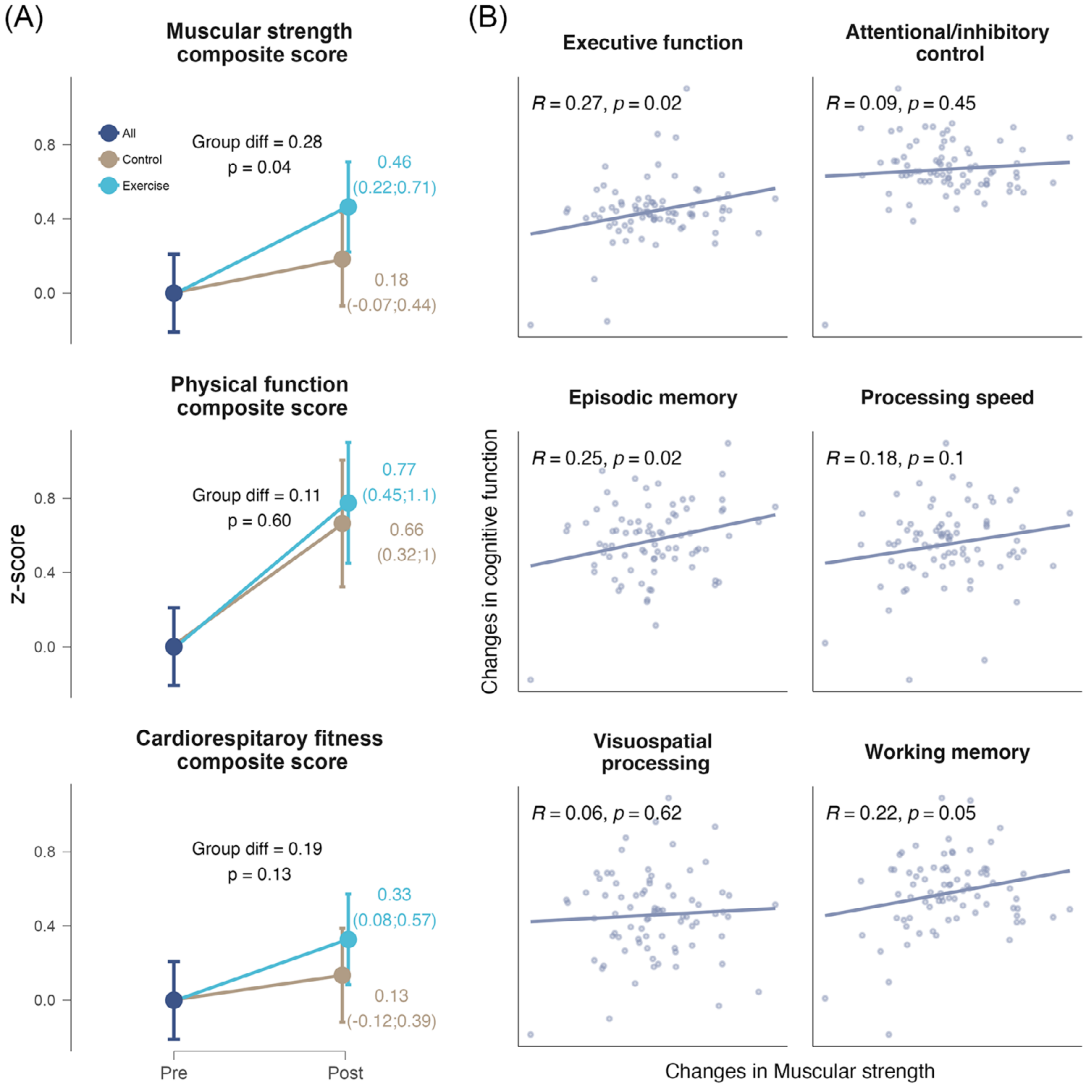
Intention‐to‐treat effects of the 24 week resistance exercise intervention on physical condition parameters (A) and correlation of muscular strength changes with cognitive function changes (B). A, Dots indicate estimated marginal means at each time point (and 95% confidence intervals). B, Dots indicate individual raw changes in each variable.

The RE effect on muscular strength, physical function, and cardiorespiratory fitness is shown in Figure 4A. Although the exercise group improved more in the three scores compared to the control group, there were only significant differences between groups on the muscular strength for both ITT (SMD = 0.28 *z* score, *p* = 0.04) and PP (SMD = 0.28 *z* score, *p* = 0.05) analyses.

### 3.6 Post hoc mediation analysis and correlation between changes

Because we detected a significant effect of the exercise intervention on muscular strength, we performed a mediation analysis to test whether changes to cognitive function were statistically mediated by changes in muscular strength score. Although no significant indirect effects was identified (Supplementary Material 7, Figure S3 in supporting information), changes in muscular strength were positively correlated with changes in EF (*R* = 0.27, *p* = 0.02), episodic memory (*R* = 0.25, *p* = 0.02), and working memory (*R* = 0.22, *p* = 0.05), but not for the other cognitive domains (all *p* ≥ 0.1; Figure 4B).

### 3.7 Intervention attendance and intensity

**FIGURE 5:**
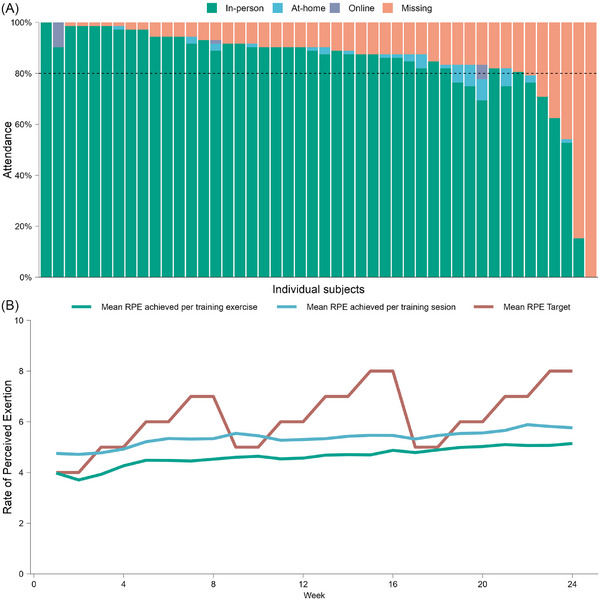
(A) Attendance and (B) intensity of the Active Gains in Brain Using Exercise During Aging (AGUEDA) resistance exercise intervention. RPE, rate of perceived exertion

In the RE intervention group (*n* = 46), mean attendance was 85.2% (83.4% in person, 1.8% online). Moreover, 10% (53 of 494 sessions) of the missed sessions were rescheduled. Two participants had < 60% attendance, four participants between 60% and 80%, and forty participants (87%) achieved > 80% of attendance (Figure 5A). The mean RPE achieved was 5.3 (range: 4.7–5.8) per session and 4.6 (range: 3.8–5.4) per exercise (Figure 5B).

### 3.8 Sensitivity analysis

Sensitivity analyses of EF, including 12 week measurements, showed no significant differences at 24 weeks (SMD = 0.13, 95% CI −0.16, 0.42), (*p* = 0.37); Supplementary Material 7, Figure S4 in supporting information). The PP analysis, restricted to participants with > 80% attendance (*n* = 84), showed similar results on cognitive outcomes (Supplementary Material 7, Figure S5 in supporting information). In this analysis, the RE group showed significantly greater attention/inhibitory control than the control group at 24 weeks (SMD = 0.41, *p* = 0.01). Sensitivity analyses with winsorized variables showed consistent findings (Supplementary Material 7, Figure S6 in supporting information).

### 3.9 Adverse events

A total of 39 AEs were reported during the 24 week intervention period, including 23 mild events (21 exercise, 2 control), 11 moderate (9 exercise, 2 control), and 5 severe (1 exercise, 4 control). All severe AEs were unrelated to the intervention. In the RE group, one participant experienced a humerus fracture. In the control group, severe AEs included esophageal surgery, pacemaker implantation, ischemic stroke, and cancer. The latter two cases were excluded from the AE statistical analysis due to their potential impact on the primary outcome, with results remaining unchanged (data not shown). The first two severe AEs occurred early in the intervention and did not interfere with the assessment procedures. Supplementary Material 7, Table S14 in supporting information, presents all AEs that occurred during the AGUEDA program.

## 4 DISCUSSION

The AGUEDA trial evaluated the effects of a 24 week RE intervention on cognitive function in cognitively normal older adults. The ITT analysis showed no group differences in EF at 24 weeks. However, the RE group demonstrated greater improvements in attentional/inhibitory control than the control group, with no effects found in the other subdomains. Exploratory analyses revealed a medium‐sized improvement in EF for those with high SCD levels and a small improvement for those with fewer years of education and the oldest adults. The RE also improved muscular strength compared to controls, which correlated positively with changes in EF, episodic memory, and working memory.

The RE produced selective improvements in attentional/inhibitory control (i.e., Flanker Test, Stroop Test, DCCST, and TMT). Previous RCTs have reported positive effects of RE on EF,^12^, ^52^ episodic memory,^53^ working memory,^54^ or general cognition, using elastic bands and single cognitive tests as outcomes.^19^, ^55^, ^56^ Our results did not corroborate these findings, suggesting that discrepancies may reflect differences in intervention protocols or participant characteristics. Systematic reviews^12^, ^23^, ^57^, ^58^ have highlighted key limitations in detecting RE's cognitive benefits, notably the unclear identification of neuropsychological tests and domains assessed. Further complexity arises from variability in EF and other domain measurements across scales, due to heterogeneous assessment methods.^59^, ^60^ Neurobiological mechanisms unrelated to the specific cognitive demands of RE may also influence outcomes.^23^ Finally, the lack of standardized reporting on exercise intervention characteristics hinders accurate comparisons across RCTs, including differences between elastic bands and traditional weight training.^59^

To note, in line with our results, some studies have found the most consistent benefits of RE linked to inhibitory control.^20^, ^61^, ^62^, ^63^ The selective cognitive improvements of attention/inhibitory control could be attributed to the dual physical and cognitive demands of RE.^64^, ^65^ In this context, Liu‐Ambrose et al.^66^ demonstrated that a 12 month RE intervention led to improvements in inhibitory processes, which were accompanied by functional changes in cortical hemodynamic activity during a Flanker task. Specifically, activation changes were observed in regions such as the left anterior insula and the middle temporal gyrus, suggesting a potential biological mechanism underlying RE‐related neuroplasticity.^66^ Second, RE may selectively enhance cognitive abilities that are most engaged during the exercises,^23^ such as sustained attention to movements or effort adjustment. For example, managing fatigue, controlling intensity, or maintaining balance and coordination with elastic bands requires inhibitory regulation, potentially leading to domain‐specific cognitive gains.^67^ RE also demands the conscious inhibition of automatic responses (e.g., ensuring proper technique), a core aspect of inhibitory control.^68^ Emerging research suggests that coordination or cognitive‐motor training linked to RE promotes neuroplasticity.^65^, ^69^, ^70^ These approaches involve motor tasks that require planning, sequencing, and coordination of multiple body parts, skills closely tied to inhibitory control processes.^71^ In contrast, other cognitive functions, such as working memory or processing speed, may be more sensitive to aerobic exercise, which involves more passive cognitive engagement (e.g., walking). In line with this, a previous systematic review^24^ emphasized the importance of identifying the cognitive domains most sensitive to exercise‐induced improvements.

Exploratory analyses, although hypothesis generating and requiring careful interpretation, indicated considerable effects on EF in subgroups with lower education backgrounds, older age, and higher SCD. The severity of self‐reported memory complaints also appeared to moderate the effects of RE on attentional/inhibitory control, processing speed, and working memory. These findings are consistent with prior research showing greater responsiveness to exercise interventions, including RE, in high‐risk populations for cognitive decline (e.g., oldest participants, ≥ 80 years,^34^ those with lower education levels, or those with higher SCD ^28^). Furthermore, our results reinforce the idea that SCD itself may serve as a preclinical marker for identifying individuals who are particularly responsive to interventions targeting cognitive decline.^72^, ^73^ Previous research has both supported and questioned the direct interaction between exercise and SCD‐related cognitive changes,^74^ though early intervention in SCD may improve the functioning and slow cognitive decline.^75^ In our study, cognitive performance did not differ among SCD groups; however, participants with higher SCD showed significantly higher brain amyloid beta levels. Speculative hypotheses may explain why individuals with greater SCD derive greater benefit from exercise. First, exercise enhances cognitive reserve, neural plasticity,^76^ and increases brain volume,^77^ thereby improving neural efficiency in individuals whose cognitive reserve is more vulnerable (i.e., those with higher SCD) and modifiable through factors such as education.^78^ Second, exercise promotes social engagement, emotional stability, and reduced distress,^79^ which is particularly relevant for individuals with SCD, who often experience social withdrawal.^80^ Third, exercise may help compensate for less efficient cognitive processing in high‐SCD individuals by enabling more effective allocation of cognitive resources.^28^, ^74^ Collectively, this evidence highlights the potential of personalized exercise interventions tailored to individual characteristics, such as those with memory complaints, even before reaching a clinical phase.

Demonstrating the efficacy of a RE intervention requires evidence of improvements in muscular strength, the primary physiological target. In this study, the AGUEDA RE intervention elicited the most robust effects on muscular strength. Previous meta‐analyses have repeatedly shown that elastic‐band RE enhances muscular strength^81^, ^82^ and have also identified upper‐body measures, including handgrip strength, as a potential marker for monitoring cognitive changes and predicting cognitive decline.^83^, ^84^ Our findings support this association, revealing positive correlations between muscular strength improvements and EF, episodic memory, and working memory. Although these findings may underscore the potential role of muscular strength as a determinant of cognitive health,^85^ no mediating effect was found in our analyses. Future larger RCTs using elastic bands RE are needed to clarify the mediating pathways in mediation.

Exercise‐related parameters are crucial components of interventions’ efficacy.^4^ In the AGUEDA intervention, the exercise group received a relatively higher than usual attendance, as evidenced by an average rate of > 85% of the total prescribed sessions (61.2 out of 72 hours), compared to previous reports with an average attendance rate of 70% (CI: 69%–73%).^86^, ^87^ More than 87% of participants achieved the predefined PP threshold of 80% attendance. The average session intensity (RPE 5.4/10) was within the prescribed range (4–8).^40^ Attendance and intensity likely contributed to the observed effects, consistent with evidence linking training volume to improvements in inhibitory control.^88^ In this context, ours‐Osman et al. suggested that at least 52 h of exercise (e.g., RE and others) may be required to improve cognitive performance, particularly in attentional/inhibitory control, in older adults.^24^ The undetectable EF improvements in the AGUEDA RE group at 12 weeks may reflect insufficient exposure (36 hours of exercise), whereas a greater chance of gains emerged at 24 weeks (72 hours prescribed; 61.2 hours completed). These findings suggest that certain cognitive domains may require longer duration interventions to achieve meaningful improvements.^24^, ^60^ Moreover, subgroup effects that showed trends toward significance in this study may potentially be confirmed in future adequately powered trials with different exercise characteristics.

This study has limitations. The small sample size reduced power for moderation and mediation analyses. Recruitment during the COVID‐19 pandemic may have influenced the results, although exclusion criteria prevented enrolling participants with prior severe COVID‐19. The absence of validated objective methods to quantify external load with elastic bands^81^ is another limitation which we intend to address in future research using recently proposed load quantification equations.^89^ AEs were documented retrospectively, potentially impairing the accuracy of reported incidents. Increased contact with the research team in the RE group may have led to a greater detection of mild and moderate AEs. However, this study had several strengths, including the RCTs design and the use of elastic bands and body weight requiring minimal equipment and allowing for scalability. The program's practicality is further underscored by high adherence rates. Additionally, the comprehensive neuropsychological assessment, with multiple tests per cognitive subdomain, and the sample's exceptional phenotyping across all domains, are notable strengths of this study.

In conclusion, the AGUEDA RE intervention showed selective benefits in attentional/inhibitory control, suggesting that the cognitive effects of RE differ across cognitive domains and are influenced by participant characteristics, particularly in individuals with higher risk profiles (i.e., higher SCD). This RE intervention represents a safe, feasible, and low‐cost strategy with potential for public health applications in preserving cognitive function. Future research should explore the dose–response relationship, extend intervention duration, and determine the mechanisms underlying these selective effects to optimize tailored exercise intervention for improving cognitive health.

## CONFLICTS OF INTEREST STATEMENT

The authors declare that they have no conflicts of interest. The sponsors had no role in the study design and conduct, data collection, analysis, and interpretation, manuscript preparation, or review or approval of the manuscript. Author disclosures are available in the supporting information.

## CONSENT STATEMENT

All human subjects provided informed consent.

## Supporting information



Supporting Information

Supporting Information
